# Genotyping and zoonotic potential of *Enterocytozoon bieneusi* in cattle farmed in Hainan Province, the southernmost region of China

**DOI:** 10.1051/parasite/2020065

**Published:** 2020-11-24

**Authors:** Xin-Li Zheng, Huan-Huan Zhou, Gangxu Ren, Tian-Ming Ma, Zong-Xi Cao, Li-Min Wei, Quan-Wei Liu, Feng Wang, Yan Zhang, Hai-Long Liu, Man-Ping Xing, Li-Li Huang, Zhe Chao, Gang Lu

**Affiliations:** 1 Institute of Animal Science and Veterinary Medicine, Hainan Academy of Agricultural Sciences 571100 Haikou PR China; 2 Key Laboratory of Tropical Translational Medicine of the Ministry of Education, Hainan Medical University 571199 Haikou PR China; 3 Department of Pathogenic Biology, Hainan Medical University 571199 Haikou Hainan PR China; 4 Hainan Medical University-The University of Hong Kong Joint Laboratory of Tropical Infectious Diseases, Hainan Medical University 571199 Haikou Hainan PR China

**Keywords:** *Enterocytozoon bieneusi*, Cattle, Genotyping, Hainan (China)

## Abstract

*Enterocytozoon bieneusi* is an intestinal pathogen that infects a wide range of species, including humans. Cattle constitute an important host for *E. bieneusi*; however, there is a scarcity of information on the prevalence and genotyping of *E. bieneusi* in cattle in the Hainan Province of China. In this study, PCR analysis of 314 fecal samples from cattle in six cities of Hainan was performed for genotype identification. The average prevalence of *E. bieneusi* in these animals was 9.9% (31/314), and ranged from 0.0% (0/12) to 20.5% (8/39). Five known genotypes – EbpC (*n* = 14), BEB4 (*n* = 12), J (*n* = 2), I (*n* = 1), and CHG5 (*n* = 1) – and a novel genotype: HNC-I (*n* = 1) – were identified. Genotypes EbpC and HNC-I were placed in zoonotic Group 1, and the remaining four genotypes (BEB4, J, I, and CHG5) were placed in Group 2. Since 93.5% of the genotypes found in the cattle (29/31) (EbpC, BEB4, J, and I) have previously been found in humans, these genotypes are probably involved in the transmission of microsporidiosis to humans.

## Abstract (fr)

*Enterocytozoon bieneusi* est un pathogène intestinal qui infecte un large éventail d’espèces, y compris les humains. Le bétail constitue un hôte important pour *E. bieneusi*, mais les informations sur la prévalence et le génotypage d’*E. bieneusi* chez les bovins de la province de Hainan en Chine sont rares. Dans cette étude, une analyse PCR de 314 échantillons fécaux provenant de bovins dans six villes de Hainan a été réalisée pour l’identification du génotype. La prévalence moyenne d’*E. bieneusi* chez ces animaux était de 9,9 % (31/314), et variait de 0,0 % (0/12) à 20,5 % (8/39). Cinq génotypes connus, EbpC (*n* = 14), BEB4 (*n* = 12), J (*n* = 2), I (*n* = 1) et CHG5 (*n* = 1), et un nouveau génotype, HNC-I (*n* = 1), ont été identifiés. Les génotypes EbpC et HNC-I sont placés dans le groupe zoonotique 1, et les quatre génotypes restants (BEB4, J, I et CHG5) sont placés dans le groupe 2. Puisque 93,5 % (29/31) (EbpC, BEB4, J et I) des génotypes trouvés chez les bovins ont déjà été trouvés chez l’homme, ces génotypes sont probablement impliqués dans la transmission de la microsporidiose à l’homme.

## Introduction

*Enterocytozoon bieneusi*, a zoonotic intestinal pathogen, infects a wide range of species worldwide [20, 24]. Microsporidiosis occurs through the ingestion of infectious spores of *E. bieneusi* through contaminated soil, feces, surfaces, water, as well as by improper farming practices, such as using untreated animal manure as fertilizer directly on open crops or tillage land [20]. *Enterocytozoon bieneusi* has received considerable attention due to its known propensity to cause both water- and food-borne outbreaks of illness [44].

Sequence analysis of the internal transcribed spacer (ITS) region of the ribosomal RNA (rRNA) gene has revealed more than 500 genotypes (142 in humans, of which 49 were also identified in animals) [11, 20, 54]. Phylogenetic comparative analyses clustered all genotypes into eleven major genetic groups. Human cases have been reported to show infection with *E. bieneusi* genotypes from six groups, and more than 90% of human-pathogenic genotypes belonged to Group 1 or Group 2 [20, 54].

Thirty-eight studies from 14 countries have identified more than 80 genotypes in cattle, known carriers of *E. bieneusi* (Table 1). Among them, at least 17 genotypes (BEB4, BEB6, I, J, PtEb XI, EbpC, D, EbpA, M, Type IV, Peru 6, H, O, CS-4, CHN3, CHN4, and S7) have also been identified in humans [20]. Of the remaining 67 genotypes, 30 belonged to Group 1, and 27 belonged to Group 2, indicating the vital role of cattle in the epidemiology of *E. bieneusi* and their ability to transmit the pathogen to humans [20]. Therefore, cattle infected with *E. bieneusi* may pose a threat to public health.

**Table 1:** ITS genotypes of *Enterocytozoon bieneusi* of natural infection identified in cattle worldwide.

Country	Positive/examined (%)	Genotypes^a^ (*n*)	Ref.
Algeria	11/102 (10.8)	**BEB4** (4), **BEB6** (2), BEB3 (1), **I** (1), **J** (1), **PtEb XI** (1), mixed (1)	[2]
Argentina	10/70 (14.3)	**BEB4** (1); **I** (2), **J** (4); **EbpC** (1); BEB10 (1); **D** (1)	[5]
Australia	49/471 (10.4)	**I** (18), **J** (14), **BEB4** (6), TAR_fc2 (6), TAR_fc1 (1), TAR_fc3 (1)	[48]
Brazil	79/452 (17.5)	**I** (35), BEB8 (23), **BEB4** (7), BEB13 (7),BEB12 (5), **D** (4), BEB11 (3), **EbpA** (1), BEB14–BEB17 (1 each)	[4]
China	1817/10504 (17.3)	**J** (904); **I** (519); **BEB4** (151), **BEB6** (31), **O** (27), CM8 (18), COS-I (14), **EbpC** (14), **CHN3** (14), **D** (13), **CHN1** (11), CGC3 (11), CGC2 (8), CHC8 (7), CGC1 (6), **CHN4** (6), **CS-4** (6), **Type IV** (5), CM19 (5), BEB10 (3), CHG2 (2), CHG3 (2), CHN–DC1 (2), CHN–DC2 (2), CHN–DC3 (2), G (2), NECA1–NECA5, CHC1–CHC7, CHC9–CHC17, **N**, BEB8, CD6, **H**, CC4, CSX1–CSX2, CHN15, CM21, PN, and mixed (1 each)	[10, 12, 13, 19, 23, 26, 37, 38, 41–43, 46, 47, 49, 50]
Czech Republic	37^b^/240 (15.4)	**I** (6)	[14]
Germany	10/88 (11.4)	**I** (2); **J** (4); **F** (1); **M** (1); **N** (1), **I/J** (1)	[6, 27]
Iran	48/256 (18.8)	**D** (22), **J** (9), **M** (5)	[16]
Korea	80^b^/538 (14.9)	**CEbE** (3), CEbD (2), **CEbB** (2), CEbA (1), CEbF (1), **CEbC** (1)	[17]
Portugal	2/2^c^ (100.0)	**PtEbX** (1), **PtEbXI** (1)	[22]
South Africa	9/50 (18.0)	**BEB4** (3); **I** (1); BEB3-like (4); **D** (1)	[1]
United States	706/3306 (21.4)	**J** (110); **BEB4** (120); **BEB**2 (85); **I** (79), **BEB**1 (47); BEB8 (41); BEB5 (8); BEB9 (6); BEB3 (6); **Peru 6** (1); D (1), BEB7 (1), **Type IV** (1)	[8, 9, 31–33, 36]
Slovakia	2/100 (2.0)	**I** (2)	[40]
Thailand	3/60 (5.0)	**D** (3)	[39]

a The names of genotypes are from publications.

b The number of genotypes is not consistent with the number of positives because only some *E. bieneusi* isolates were genotyped in the Czech Republic and Korea.

c Only two isolates positive for *E. bieneusi* by microscopy after staining were genotyped in Portugal.

The genotypes previously found in humans are shown in bold.

In China, cattle farming and dairy products are important economic industries. Previous studies on *E. bieneusi* in cattle in China focused on inland cities and did not include assessments in Hainan Province, the southernmost region of China, where, local yellow cattle breeding is very popular. Here, we evaluated the prevalence, genetic characteristics, and zoonotic potential of *E. bieneusi* in cattle from six cities of Hainan Province.

## Materials and methods

### Ethics statement

The study was initiated after obtaining written informed consent for animal use by farm owners. All animal experiments were reviewed and approved by the Ethics Committee of Hainan Medical University.

### Fecal specimen collection

**Figure 1:**
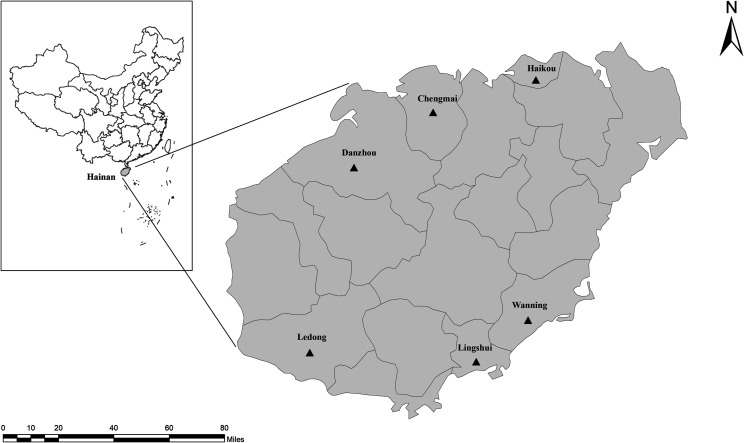
Specific locations where samples were collected in this study. ▲: Sampling points.

In all, 314 fecal samples were gathered from 10 cattle farms in six cities of Hainan Province between March and December 2019 (Fig. 1 and Table 2). The cattle farms were selected based only on the owners’ willingness to participate and the accessibility of animals for sampling. Samples were obtained from 30–50% of the total number of cattle on each farm. A sterile disposable latex glove was used to collect the fecal specimens immediately post defecation, and placed in individually labeled plastic bags. Cattle were divided into two groups: young aged ≤ 12 months (*n* = 18) and adults aged > 12 months (*n* = 296). Cattle were in good health at the time of sampling. Within 24 h of sampling, the labeled fecal bags were transported and stored in the laboratory at 4 °C and were processed within 48 h.

**Table 2:** Prevalence and genotype distribution of *E. bieneusi* isolates in cattle in Hainan Province.

Category		Positive/examined (%)	Genotype(s) (*n*)
Location	Farm 1 (Chengmai)	2/13 (15.4)	EbpC (2)
	Farm 2 (Chengmai)	0/5 (0.0)	/
	Farm 3 (Chengmai)	2/64 (3.1)	J (2)
	Subtotal (Chengmai)	4/82 (4.9)	EbpC (2), J (2)
	Farm 4 (Danzhou)	8/39 (20.5)	EbpC (7), I (1)
	Farm 5 (Haikou)	0/4 (0.0)	/
	Farm 6 (Haikou)	1/50 (2.0)	EbpC (1)
	Subtotal (Haikou)	1/54 (1.9)	EbpC (1)
	Farm 7 (Ledong)	0/12 (0.0)	/
	Farm 8 (Lingshui)	0/26 (0.0)	/
	Farm 9 (Lingshui)	12/70 (17.1)	BEB4 (12)
	Subtotal (Lingshui)	12/96 (12.5)	BEB4 (12)
	Farm 10 (Wanning)	6/31 (19.4)	EbpC (4), CHG5 (1), HNC-I (1)
Age^a^	≤12 months	4/18 (22.2)	EbpC (3), I (1)
	>12 months	27/296 (9.1)	BEB4 (12), EbpC (11), J (2), CHG5 (1), HNC-I (1)
Total		31/314 (9.9)	EbpC (14), BEB4 (12), J (2), I (1), CHG5 (1), HNC-I (1)

a All the cattle aged ≤ 12 months were from farm 4 in Danzhou city.

### DNA extraction

All fecal specimens were filtered through sieve in distilled water, followed by centrifugation at 1500 ×*g* for 10 min. A QIAamp DNA stool mini kit (QIAgen, Germany) was used to isolate the genomic DNA of each processed specimen (approximately 200 mg), following the manufacturer’s instructions. A total of 200 mL AE elution buffer was used to elute the DNA, followed by storage at −20 °C before PCR analysis.

### Polymerase chain reaction (PCR) amplification

*Enterocytozoon bieneusi*-specific nested primers and cycle parameters designed by Hamed Mirjalal were used to amplify a 410 bp sequence in the ITS region of the rRNA gene using TaKaRa Taq DNA Polymerase [25]. The PCR products were analyzed using 1.5% agarose gel electrophoresis, followed by GelRed (Biotium Inc., USA) staining.

### Nucleotide sequencing and analysis

The sequence accuracy of all *E. bieneusi*-positive PCR products (sequenced by Sangon Biotech Co., Ltd., China) was confirmed through bidirectional sequencing and the sequencing of additional PCR products. The Basic Local Alignment Search Tool (BLAST) and ClustalX 1.83 were used to compare the published GenBank sequences with the ones identified in this study to identify the genotypes of *E. bieneusi*. Genotypes that were identical to the genotypes deposited in the GenBank database were given the first published name, and those that generated ITS sequences with a single nucleotide substitution/deletion/insertion were identified as novel genotypes based on the DNA sequencing of minimum two PCR products [30]. The samples were labeled in the order of appearance by adding roman numerals after HNC (Hainan Cattle). A 243 bp part of the ITS region of the rRNA gene of *E. bieneusi* was used for naming reference, following the established nomenclature system [30].

### Phylogenetic analysis

A neighbor-joining phylogenetic tree was built using Mega X software, and the Kimura-2-parameter model with 1000 replicates to evaluate the relationship between the novel ITS genotype and the known genotypes, and to confirm the gene group designation.

### Statistical analysis

Fisher’s exact test and a Chi-square test were used to evaluate the difference in infection rates among different locations and ages, respectively, using SPSS v22.0 (IBM Corp., USA). A *p*-value < 0.05 was regarded as statistically significant.

### Nucleotide sequence accession numbers

The GenBank database accession number of the identified nucleotide sequence was MT193626.

## Results and discussion

Of the 314 fecal samples, 31 (9.9%) were *E. bieneusi*-positive, based on sequence analysis of the ITS region of the rRNA gene. A significant difference in the rate of occurrence of *E. bieneusi* was observed in cattle from the six cities (*p* < 0.05), with 20.5% (8/39) in Danzhou, 19.4% (6/31) in Wanning, 12.5% (12/96) in Lingshui, 4.9% (4/82) in Chengmai, 1.9% (1/54) in Haikou, and an absence of this parasite (0/12) in Ledong (Table 2).

Since the first report of *E. bieneusi* in calves in Germany, there have been 38 published epidemiological reports on *E. bieneusi* conducted in 14 countries, and the average infection rates in these countries range from 2.0% to 21.4% (Table 1). The infection rate of *E. bieneusi*, based on cattle from 16 provinces of China, falls in the range of 2.0–37.6% (Table 3). This study reports the occurrence of *E. bieneusi* in cattle from Hainan Province. The differences in prevalence might be related to the sensitivity and specificity of detection methods, the health status of hosts, the experimental design, the overall sample size, animal practices, and so on. Like in other animals and humans, age appears to be a significant factor affecting the occurrence of *E. bieneusi* in cattle [51]. In the present study, the prevalence of *E. bieneusi* was 22.2% (4/18) in young animals ≤ 12 months and 9.1% (27/296) in adult animals > 12 months. Although the infection rates in calves were higher than those in adults, the differences were not significant (*χ* ^2^ = 1.966, *p* > 0.05) (Table 2). A study by Ma et al. revealed *E. bieneusi* infection rates in juveniles, post-weaned calves, pre-weaned calves, and adults of 4.5% (6/134), 7.7% (8/104), 10% (1/10), and 3.9% (13/332), respectively [23]. Similarly, da Fiuza et al. reported that pre-weaned calves (27.6%, 21/76) and post-weaned calves (28.8%, 44/153) showed a higher rate of prevalence of *E. bieneusi* compared with heifers (14.1%, 12/85) and adults (1.4%, 2/138) [4]. Meanwhile, Li et al. showed that calves aged < 3 months (29.3%, 127/434) and 3–12 months (23.9%, 63/264) had higher infection rates than juveniles and adults (13.3%, 24/181) [16]. In accordance with these results, it was supposed that age was negatively correlated with the prevalence of *E. bieneusi* in cattle, probably due to the underdeveloped immune systems of the young animals.

**Table 3:** ITS genotypes of natural *Enterocytozoon bieneusi* infections identified in cattle in China.

Regions	Positive/examined (%)	Genotypes (*n*)	Ref.
Gansu	320/1414 (22.6)	**J** (155), **I** (126), CGC3 (11), CGC2 (8), CGC1 (6), **BEB4** (5), CM19 (5), BEB10 (3), CM21 (1)	[43]
Guangdong	160/1440 (11.1)	**I** (91), **J** (60), **D** (4), **BEB**4 (3), **EbpC** (2)	[43]
	61/388 (15.7)	**J** (58), **D** (4)	[10]
Hebei and Tianjin	202/1040 (19.4)	**I** (87), **J** (83), **BEB4** (18), CHC8 (7), **BEB6** (3), **N** (1), **EbpC** (1), CHC6 (1), CHC7(1)	[12]
Henan	28/44 (6.0)	**I** (16), **J** (7), **BEB4** (5)	[23]
	33/277(11.9)	**BEB6** (10), COS-1 (6), **I** (6), CHG2, CHG3, **J**, CHC9, CHC10, CHC11, CHC12, CHC13, CHC14, CHC15, and CHC16 (1 each)	[46]
Henan and Ningxia	214/879 (24.3)	**J** (77), **I** (61), CM8 (18), **BEB6** (17), **BEB4** (15), **EbpC** (6), COS-1 (5), **EbpA** (2), **D** (2), BEB8, CD6, CHC1-CHC5, CHG2, CHG3, **H**, and **O** (1 each)	[19]
Heilongjiang	31/526 (5.9)	**J** (10), **CS-4** (7), **I** (3), **BEB4** (2), **EbpC** (3), G (1), NECA1 - NECA5 (1 each)	[13]
	40/133 (30.1)	**O** (26), **EbpA** (2), **J** (2), **I** (2), CHN-DC1–CHN-DC3 (2 each), **BEB4** (1), **D** (1)	[50]
	93/321 (29.0)	**BEB4** (22), **J** (40), **I** (31)	[38]
Jiangsu	177/1366 (13.0)	**J** (144), **I** (26), **BEB4** (11), **Type IV** (1), CHC17 (1)	[42]
Jilin	35/93 (37.6)	**CHN3** (14); **CHN1** (10); **J** (9); **I** (8); **CHN4** (2)	[47]
Liaoning	1/11 (9.1)	**J** (1)	[13]
Qingha and Yunnan	10/57 (17.5)	**J** (5), COS I (3), PN (1), **BEB6** (1)	[49]
Shaanxi	39/198 (19.7)	**I** (21), **J** (16), **CHN1** (1), CSX1 (1)	[41]
	34/173 (19.7)	**I** (19), **J** (14), CSX2 (1)	[41]
Shandong	21/673 (3.12)	**J** (18), **BEB4** (2), **I** (1)	[41]
	3/148 (2.0)	**I** (1), **J** (2)	[23]
Shanghai	214/809 (26.5)	**J** (145), **BEB4** (63), **CHN4** (4), **Type IV** (4), CHN15 (1), mixed (1)	[37]
Xinjiang	85/514 (16.5)	**J** (57), **I** (19), **BEB4** (4), **D** (2), **EbpC** (2), CC4 (1)	[26]

The genotypes previously found in humans are shown in bold.

Here, we identified one novel genotype (HNC-I) and five known genotypes (EbpC, BEB4, J, I, and CHG5). The novel genotype showed high similarity to genotype EbpC (AF076042), with one base variation at position 237 (C → T). Out of the six genotypes, the most prevalent genotype was EbpC (14 specimens), which was found in four of the six locations, followed by BEB4 (12 specimens), but this genotype was only found in Wanning. Genotype J was found in two cattle from Chengmai. The remaining three genotypes I, CHG5, and HNC-I were found in a single specimen, with the former from Danzhou and the latter two from Wanning. These results differed from those reported from the other regions of China. For example, in Gansu, Guangdong, Henan, Ningxia, Jiangsu, Shaanxi, and Xinjiang provinces, genotypes J and I were reported to be the dominant genotypes, and in Heilongjiang, genotype O was dominant (Table 3). Meanwhile, region-specific difference in genotype constitutions of *E. bieneusi* can also be observed in cattle in some studies, such as genotype D in Iran [16]. Therefore, the genotype distributions of *E. bieneusi* in cattle differed by region, but the reason behind this phenomenon is unclear.

In the present study, human-pathogenic genotypes EbpC, BEB4, J and I were observed with high occurrence (93.5%, 29/31). Genotype EbpC has been detected in humans, such as in cancer patients in Iran [25], in immunocompetent patients in the Czech Republic [29], in children in Peru and China [3, 45], and in HIV-positive patients in Peru, China, Iran, Thailand, and Vietnam [7, 18, 21, 25, 35, 41]. It was also found in more than 15 animal species and water samples [20]. Likewise, genotypes BEB4, J, and I were also found in humans [28, 47], non-human primates [15, 46], and other animals [20], and they have been documented in cattle (Table 1). This suggests that cattle infected with genotypes EbpC, BEB4, J, and I may facilitate transmission to other animals and humans.

The remaining genotype CHG5 and the novel genotype HNC-I were first identified in cattle here. Genotype CHG5 has been reported in goats with a wide distribution in China [34, 53]. We also observed this genotype in the Asiatic brush-tailed porcupines in Hainan Province [52]. Thus, the detection of the same genotype (CHG5) in multiple species (cattle, goats, and rodents) in the same region (Hainan, China) suggests a vast host range along with the possibility of cross-species transmission among cattle, goats and rodents.

**Figure 2:**
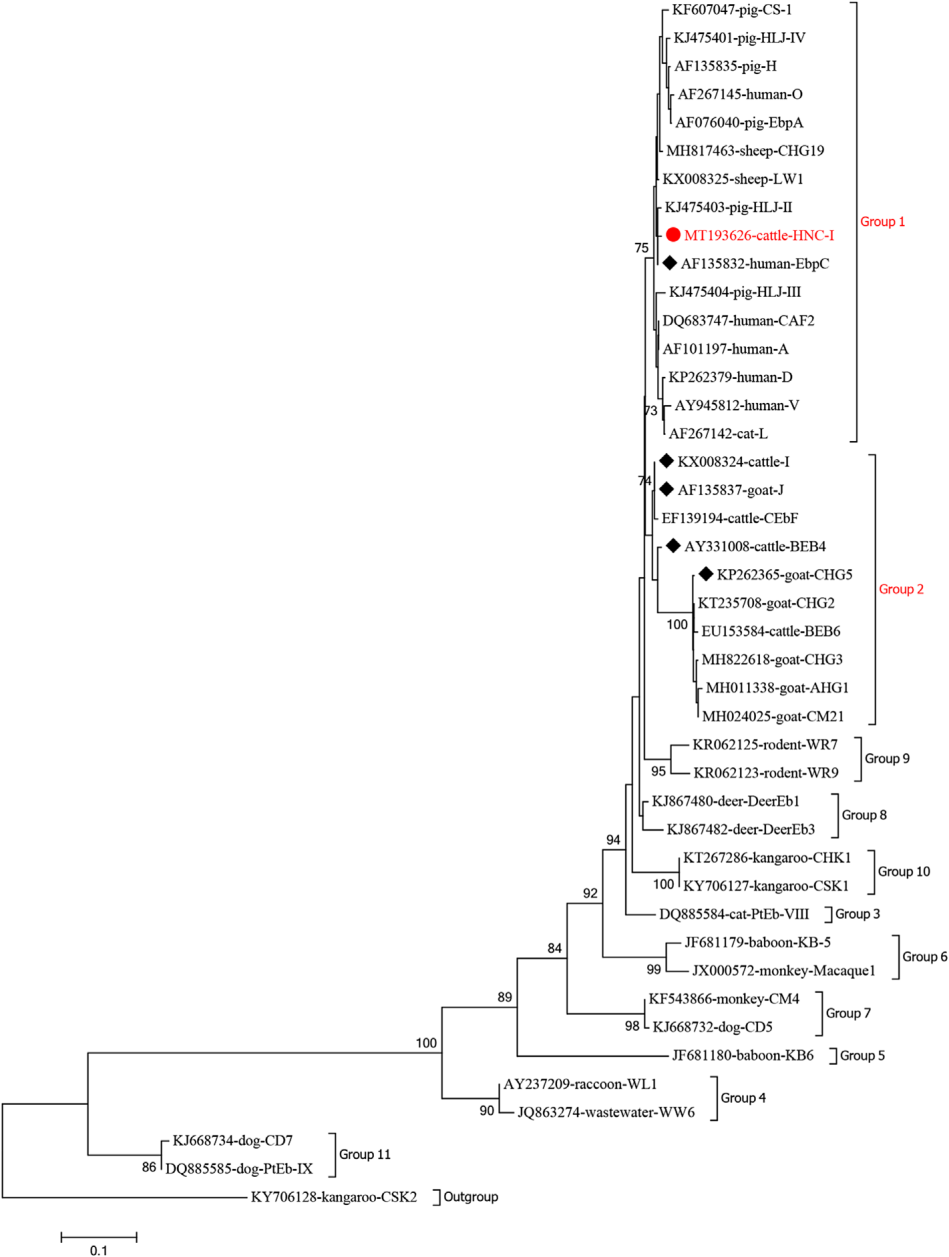
Phylogenetic tree based on neighbor-joining (N-J) analysis of ITS sequences. Phylogenetic relationships between the *E. bieneusi* genotypes identified in cattle here and other known genotypes deposited in GenBank were inferred by an N-J analysis of ITS sequences based on genetic distance by the Kimura two-parameter model. The numbers on the branches are percent bootstrapping values from 1000 replicates. Each sequence is identified by its accession number, host origin, and genotype designation. *Enterocytozoon bieneusi* genotype CSK2 (KY706128) was used as the outgroup. The squares and triangles filled in black indicate novel and known genotypes identified in this study, respectively.

The phylogenetic analysis revealed that EbpC and HNC-I, identified in this study, were divided into zoonotic Group 1, whereas genotypes BEB4, J, I, and CHG5 belong to Group 2 (Fig. 2). In total, 94.0% (79/84) of the genotypes identified in cattle clustered into Group 1 or 2 (except for genotypes CX1, CX2, TAR_fc3, CAM2, and S7) [20]. These findings suggest that *E. bieneusi*-infected cattle represent a potential threat to humans.

## Conclusion

This study is the first evaluating the infection rates, genotype characteristics, and zoonotic potential of *E. bieneusi* in cattle from Hainan Province. Our results revealed a prevalence rate of 9.9% (31/314) for *E. bieneusi* within five of six cities in Hainan, China. We identified five known genotypes and a novel genotype. Genotype EbpC and novel genotype HNC-I were grouped into zoonotic Group 1, while genotypes BEB4, J, I and CHG5 were placed in Group 2. The observed high occurrence (93.5%, 29/31) of zoonotic genotypes (EbpC, BEB4, J, and I) emphasizes the possible role of cattle in the transmission of *E. bieneusi* to humans, which requires further investigations to reduce the threats posed by these animals to public health.
